# Protocol for a mixed methods process evaluation for a randomised controlled trial to improve shared decision-making about, and uptake of, osteoporosis medicines: the iFraP study

**DOI:** 10.3310/nihropenres.13751.3

**Published:** 2026-04-02

**Authors:** Laurna Bullock, Andrea Cherrington, Emma M Clark, Jane Fleming, Ida Bentley, Elaine Nicholls, David Webb, Jo Smith, Sarah Bathers, Sarah Lewis, Robert Horne, Terence W O'Neill, Christian D Mallen, Clare Jinks, Zoe Paskins

**Affiliations:** 1 Centre for Musculoskeletal Health Research, School of Medicine, Keele University, Newcastle under Lyme, England, UK; 2 Keele Clinical Trials Unit, Keele University, Newcastle under Lyme, England, UK; 3 Bristol Medical School, Faculty of Health Sciences, University of Bristol, Bristol, UK; 4 Cambridge Public Health, University of Cambridge, Cambridge, UK; 5 Addenbrooke’s Hospital Fracture Liaison Service, Cambridge University Hospitals NHS Foundation Trust, Cambridge, England, UK; 6 School of Medicine Research User Group, Keele University, Newcastle under Lyme, England, UK; 7 Centre for Behavioural Medicine, UCL School of Pharmacy, University College London, London, England, UK; 8 Centre for Epidemiology Versus Arthritis, The University of Manchester, Manchester, England, UK; 9 NIHR Manchester Biomedical Research Centre, Manchester University NHS Foundation Trust, Manchester, England, UK; 10 Haywood Academic Rheumatology Centre, Midlands Partnership University NHS Foundation Trust, Stoke-on-Trent, UK

**Keywords:** Shared decision-making, decision aid, osteoporosis, randomised controlled trial, Fracture Liaison Service, iFraP, process evaluation, implementation

## Abstract

**Background:**

High quality shared decision-making (SDM) conversations involve people with or at risk of osteoporosis and clinicians working together to decide, where appropriate, which evidence-based medicines best fit the person’s life, beliefs, and values. The **i**mproving uptake of **Fra**cture **P**revention drug treatments (iFraP) intervention comprises a computerised Decision Support Tool (DST), clinician training package and information resources, designed for use in UK Fracture Liaison Service (FLS) consultations. The iFraP intervention will be tested in a pragmatic, parallel-group, individual randomised controlled trial in patients referred to four FLSs in England. This mixed methods process evaluation aims to assess which components of iFraP were delivered and how (fidelity), whether iFraP results in a change in osteoporosis drug treatment initiation rates and how, and how context affects implementation of iFraP and outcomes.

**Methods:**

We will collect quantitative data using (1) Case Report Forms completed by FLS clinicians; (2) self-reported questionnaires completed by patient participants; and (3) DST analytic data. We will collect qualitative data using (1) semi-structured interviews with patients who receive the iFraP intervention in their FLS appointment, FLS clinicians delivering iFraP appointments, and primary care clinicians that have consulted with a patient following their iFraP FLS appointment; and (2) FLS consultation recordings. A triangulation protocol will be used to integrate the quantitative and qualitative findings to generate novel insights about the intervention under evaluation.

**Discussion:**

The process evaluation, alongside the trial, will help to understand what elements of the iFraP intervention were delivered and how, the mechanisms of impact and how context affected implementation and outcomes, and intervention acceptability. Mixed methods interpretation will lead to further insights about the implementation of SDM and DSTs in-practice.

**Trial registration:**

**ISRCTN:** 10606407, 21/11/2022 https://doi.org/10.1186/ISRCTN10606407

## Plain Language Summary

**Background:**

Fragility fractures (or broken bones) can result in significant disability and loss of independence and confidence. Many people who have osteoporosis are not offered, do not start, or do not continue to take medicine to reduce the chance of fractures. Sometimes this is because people with osteoporosis are concerned about the medicine side effects or are unsure about what the benefits are.

To address this, we developed the ‘iFraP intervention’:

1. The iFraP ‘decision support tool’: to support patients and healthcare professionals talk together to make decisions about osteoporosis medicines

2. iFraP training for healthcare professionals to:

a. use the tool in appointments with patients
b. give understandable, clear and consistent information
c. listen to and address patient concerns

The iFraP intervention is being tested in a randomised controlled trial to see if it improves decision-making about osteoporosis medicines. Alongside the trial, this process evaluation will answer questions, such as:

- How do healthcare professionals use the tool and training? What factors influence this?
- What do patients and healthcare professionals think of the tool?
- What do healthcare professionals think of the training?
- What parts of the tool and training work (or do not work)? What factors influence this?
- Does the tool help patients and healthcare professionals to make decisions about osteoporosis medicines, and how?

**Methods:**

We will use the range of approaches:

- Video/audio-recorded appointments
- Interviews with patients and healthcare professionals
- Questionnaires completed by patients
- Information about if, and how, the tool is used in consultations

Findings from each approach will be combined to learn about the iFraP intervention.

**Outputs:**

This process evaluation will help to understand how the iFraP intervention was used and why, how the intervention works (or not), and factors that influence this. Findings will help to learn about how decision support tools can be used in practice.

## Introduction

### Background and rationale

Shared decision-making involves the patient and clinician working together to make decisions based on evidence (including risks, benefits, and possible treatment options) and the patient’s life, preferences, beliefs, and values. ^1^ For people with osteoporosis and/or high fracture risk, evidence-based medicines, such as bisphosphonates, are recommended by the National Institute for Health and Care Excellence (NICE). ^2^ Despite being inexpensive, cost-effective, readily available and effective at reducing fracture risk, 25% of people who are offered medication decline it (non-initiation). ^3^ Among those who are recommended osteoporosis medicines at specialist Fracture Liaison Services (FLSs; sometimes referred to as Fracture ‘Prevention’ Services), few persist for long enough for it to be effective, with persistence estimated at 28% at one-year post-fracture. ^4^ Low levels of osteoporosis medicine initiation and persistence, collectively described as ‘adherence’, ^5^ are optimised if a person believes that a medicine is necessary, relevant, safe, and practicable. ^6^ This demonstrates how shared decision-making has the potential to support osteoporosis medicine adherence, ^7, 8^ by ensuring that the medicine is a good ‘fit’ for the patient. ^9^

NICE’s shared decision-making guidelines recommend that, where available, clinicians should use ‘tools’ to implement shared decision-making – often called decision aids (DAs), conversation aids, or decision support tools (DSTs) - as one part of a ‘toolkit’ alongside other clinician skills. ^1^ DSTs are a well-recognised mechanism to support shared decision-making, ^7^ however, several unanswered questions remain regarding the effectiveness and implementation of DSTs. Further research has been called for to explore if and how DSTs promote meaningful patient involvement in healthcare decisions, ^10^ highlighting the need to further investigate the mechanisms of impact that underpin the effectiveness of DSTs. Furthermore, despite availability of many DSTs ^7^ (including osteoporosis DSTs ^11^), the existence of high-quality evidence supporting the use of DSTs to facilitate shared decision-making, ^7^ and shared decision-making being an NHS public health priority, ^12^ implementation of DSTs in clinical practice is poor. ^13^ A recent survey found that 79% of patient DSTs were not implemented into clinical practice following published randomised controlled trials, ^13^ with limited research providing an in-depth investigation of DST implementation. ^14^ Barriers to implementation that require further exploration include: low perception of quality and maintenance of DSTs; limited awareness, knowledge, and skills to use DSTs; perception that ‘we do shared decision-making already,’ or that ‘patients don’t want shared decision-making’; usability of DSTs with diverse patients; and failing to fit DSTs into the clinical structure or ‘workflow’ alongside competing demands and priorities. ^15, 16^ Many of these barriers may be linked to the minimal or complete lack of training provided to clinicians to implement DSTs in clinical practice, ^17^ with DSTs often described as ‘requiring minimal training for use’. However, to promote successful implementation of shared decision-making in routine clinical practice, it is important those delivering the DST ‘buy in’ to shared decision-making and are provided with adequate shared decision-making skills training. ^18^

Remote consultations became more commonplace in the context of COVID19, ^19^ accelerating what was already an NHS England priority. ^20^ However, there is concern that remote consultations, without proactive efforts to ensure equity, are associated with lower shared decision-making. ^21^ Importantly, this would be particularly detrimental to people with existing health disparities, with qualitative research during the pandemic identifying that some patients lack confidence consulting by telephone, likely detrimental to shared decision-making. ^22, 23^ This is despite evidence suggesting that shared decision-making interventions, such as DSTs, are of more benefit to disadvantaged groups and are therefore a potential mechanism by which to reduce health inequalities. ^24^ Limited research to date has evaluated the quality of shared decision-making or the use of DSTs in remote consultations. ^25^

### The iFraP study

The iFraP randomised controlled trial examines the effectiveness of the iFraP intervention compared with usual FLS practice and is the subject of this process evaluation. Protocols outlining the iFraP intervention development and randomised controlled trial are published elsewhere. ^26, 27^ This paper will therefore only briefly describe the iFraP intervention and trial to give context to the process evaluation design.

The context for the iFraP intervention and trial are FLSs in England, UK. FLSs enact secondary fracture prevention by systematically identifying adults aged ≥50 years with fragility fractures and conducting bone health assessments. Services are usually nurse or allied health professional-led and address bone health by assessing the patient’s risk of falls and future fracture and providing treatment recommendations to the patient and primary care, at one or more consultations, typically 2 months after the fracture.

The iFraP intervention aims to improve patient ease in decision-making about osteoporosis medicines (by increasing the extent that the patient was informed and involved in the consultation), to facilitate shared decision-making. It consists of three core components:

1. The iFraP DST, used on the computer during a face-to-face or telephone FLS consultation. The DST includes clinician decision-support and a patient-facing DA. It is dynamic, interactive, and tailored to the risks and needs of the patient.
2. iFraP Enhanced Consultation Skills Training Course, completed by FLS clinicians. The course includes( 1) an interactive eLearning package including modules introducing the intervention and guidance on using the iFraP DST in-practice, risk communication techniques, shared decision-making skills, universal precautions for health literacy and communicating about osteoporosis; and( 2) one 3-hour role play session, facilitated by experts in osteoporosis, shared decision-making and consultation communication skills.
3. Information resources (paper and online) for the patient and primary care clinician e.g. General Practitioner (GP) to refer to after the FLS consultation. This includes an individualised A4 PDF output (described as the ‘personal Bone Health Record’) from the iFraP DST. The Bone Health Record is accompanied by a dentist card that the patient can show to their dentist to support conversations about osteoporosis medicine and dental care.

The iFraP intervention is being tested in4 FLSs in England in Stoke-on-Trent, Portsmouth, Wolverhampton and Oxford that decide, recommend, and communicate osteoporosis medicine recommendations to patients. FLS models of care and service provision varies across the UK and consultations are enacted using different communication modalities (remote or face-to-face), ^28^ demonstrating important contextual factors that may influence the implementation of shared decision-making and a DST.

### Mixed methods process evaluation

Randomised controlled trials often test the effectiveness of multicomponent complex interventions in multisite, pragmatic trials, where there is likely to be variation in how the ‘same’ intervention is implemented. A mixed methods process evaluation collects and combines quantitative and qualitative data to deepen and broaden understanding about the inherent complexity of a complex intervention delivered in complex systems. Mixed methods process evaluations, alongside randomised controlled trials, expand the question from “is the intervention effective?” to “effective for whom, under what circumstances, and why?”, ^29^ to address the evidence gap about shared decision-making implementation in different clinical contexts. ^25^

### Aims and objectives

This mixed method process evaluation, in line with the Medical Research Council (MRC) guidance, ^30^ aims to understand what is implemented and how, how the delivered iFraP intervention produces change (or not), and how context affects implementation of iFraP and outcomes. Specific objectives are to:

1. Investigate (quantitative) and explore (qualitative) how the iFraP intervention is delivered and received, including quantity and quality of implementation (fidelity, dose and adaptation) and explore factors influencing implementation variation, including barriers to, and facilitators of, implementation, such as perceived acceptability (qualitative)
2. Integrate the quantitative results and qualitative findings to draw inferences about the implementation of the iFraP intervention (mixed methods)
3. Investigate (quantitative) and explore (qualitative) the iFraP intervention’s hypothesised mechanisms of impact and outcomes, including contextual factors associated with variability, to test the proposed intervention theory
4. Integrate the quantitative results and qualitative findings to draw inferences about how the iFraP intervention works, including factors associated with variation (mixed methods)

### Theoretical framework

**Figure 1.**
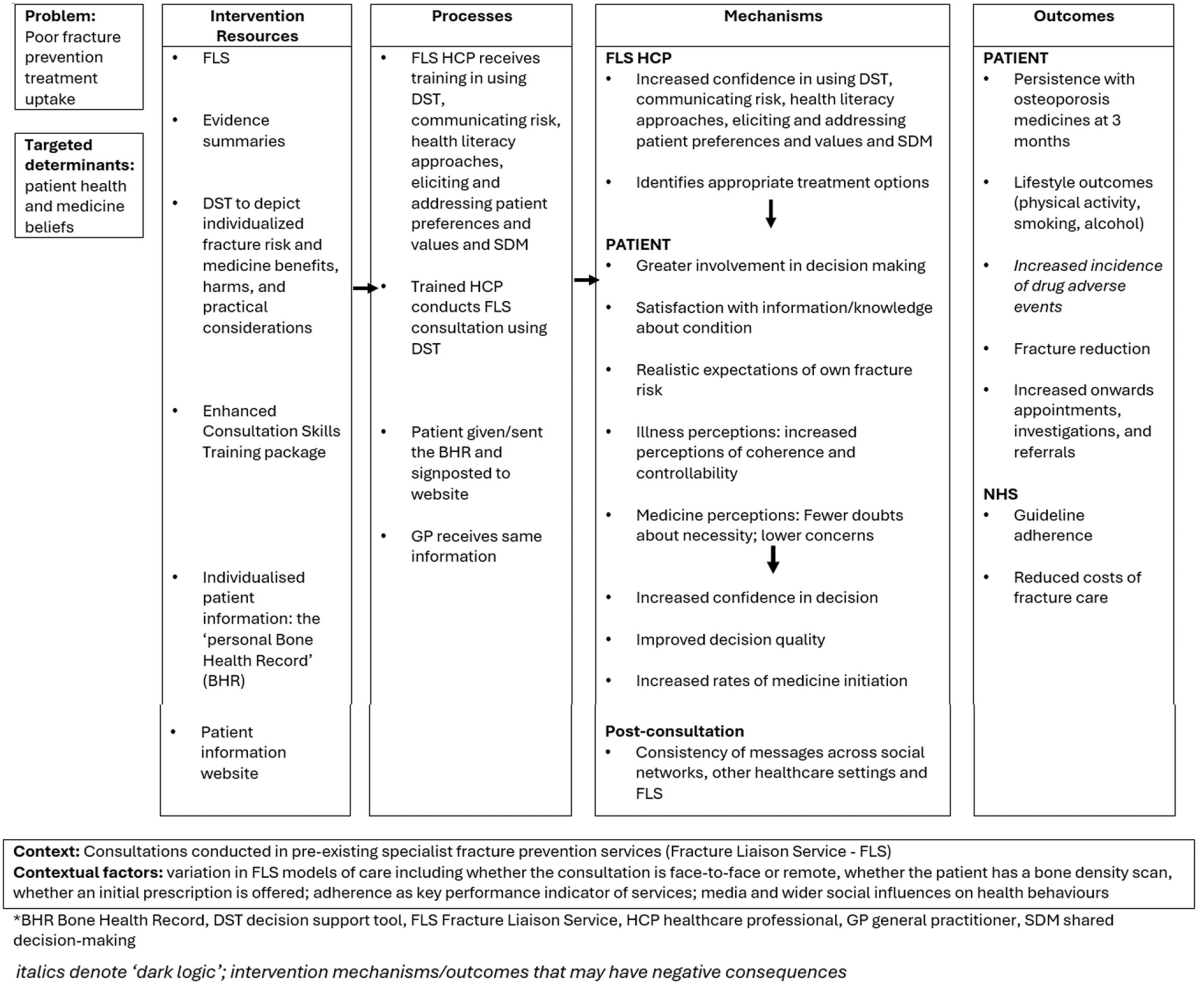
iFraP intervention programme theory.

The intervention programme theory (or ‘logic model’) details the iFraP intervention resources, hypothesised mechanisms and outcomes, and contextual factors. The iFraP programme theory was developed at the start of the iFraP study based on existing evidence and theories, and further refined iteratively throughout the intervention development work (see Figure 1).

The iFraP DST and Enhanced Consultation Skills Training Course targets FLS clinician behaviour change by increasing clinicians’ skills in shared decision-making and clinical decision-making, specifically, increasing their own confidence in communicating risks, health literacy approaches and in eliciting and addressing patient beliefs, preferences and values. By targeting clinician behaviour change, hypothesised mechanisms of action include greater patient involvement in decision-making, satisfaction with information about osteoporosis and bone health, realistic expectations of own fracture risk, and modified illness and treatment perceptions (see Figure 1). These mechanisms of action are, in part, underpinned by the Extended Self-Regulatory Model, ^31^ which incorporates both Leventhal’s Common-Sense Model of Illness ^32^ and the Necessity Concerns Framework, ^33^ acknowledging the link between illness perceptions, beliefs about medicines and medicine adherence. ^31^ Leventhal’s Common-Sense Model of Illness outlines cognitive illness representations that, together, help people to ‘make sense’ of their illness. In osteoporosis research, it’s common for people to not understand their bone health (illness coherence) ^34^ and have low perceptions about how controllable osteoporosis is (illness controllability) ^34^ e.g. thinking that osteoporosis is an inevitable part of ageing. The Necessity Concerns Framework informs the UK’s National Institute for Health and Care Excellence (NICE) guidance on medicines adherence, ^6^ postulating that when patients make decisions about taking a prescribed medicine, they weigh up their perceived need for the medicine (necessity beliefs) against concerns that they have about their medicine (concern beliefs). Evidence suggests that eliciting and addressing patient illness perceptions and beliefs about medicines as part of a shared decision-making conversation can promote medicine commitment, by ensuring that the medicine is a good ‘fit’ for the patient.

In response to intervention development work findings about the importance of having better integration between specialist (FLS) and primary and other healthcare, ^35^ the iFraP intervention resources also include an individualised patient information leaflet (known as the ‘personalised Bone Health Record’) to be given/sent to the patient and their GP after the FLS consultation, and a card which patients can show their dentist, if they are recommended osteoporosis medicines. These resources are hypothesised to improve the consistency of messages across primary care, dentistry, FLS and other social networks.

The iFraP intervention together is hypothesised to produce outcomes, including patient lifestyle changes for improved bone health and osteoporosis medicine uptake, ultimately reducing fractures.

The intervention programme theory also lists candidate contextual factors that may be associated with expected outcomes, identified in the intervention development work, including variation in FLS models of care, ^28^ adherence as key performance indicators of services, ^35^ and the influence of media and wider societal perceptions of osteoporosis on health behaviours.

## Methods

### Patient and Public Involvement and Community of Practice

To help with the development and delivery of the intervention, a group of public contributors with osteoporosis and their carers was convened. Public contributors met throughout the iFraP intervention development work and continue to meet throughout the randomised controlled trial. Involvement of public contributors throughout the iFraP study has been described elsewhere. ^26, 27^ Dedicated public involvement meetings helped to design the process evaluation and will continue to guide data collection, interpretation and dissemination.

Communities of Practice (CoPs) bring together expertise with a common concern or interest, with the aim of improving and learning to do better through regular group interaction. ^36^ iFraP CoP members include FLS clinicians, GPs, osteoporosis specialists, patients with experience of using osteoporosis medicines (supported by a public involvement worker), representatives from the Royal Osteoporosis Society (ROS) and Health Literacy UK and a behaviour change expert. The iFraP CoP met regularly throughout intervention development and will continue to meet during the iFraP trial and process evaluation (e.g. to discuss findings, knowledge mobilisation and dissemination).

### Data collection and analysis

**Figure 2.**
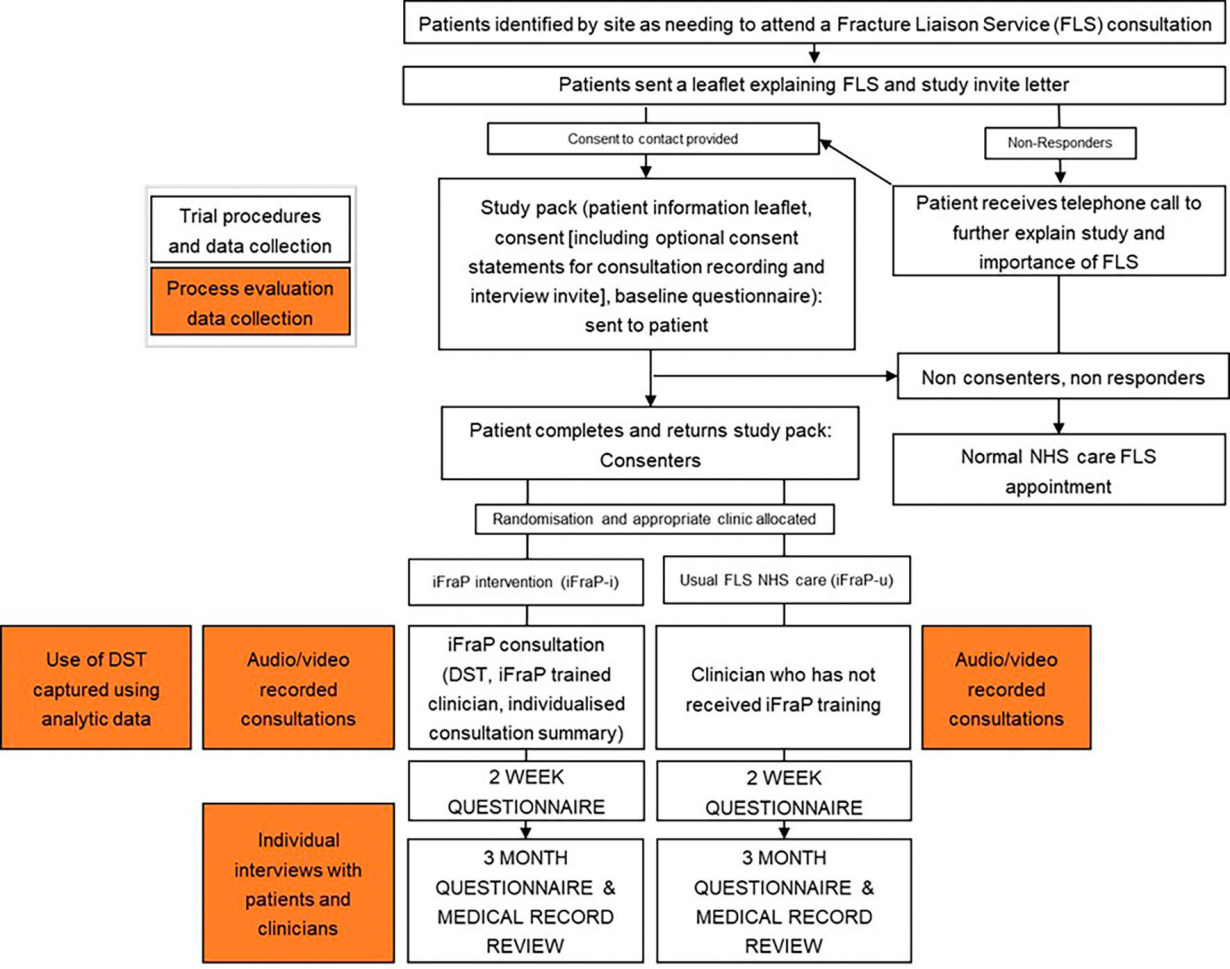
Process evaluation data collection and trial procedures.

The MRC process evaluation guidelines outline the need to be transparent about the degree of separation or integration between process and outcome evaluation teams. ^30^ In this study, the process evaluation team will work separately to prevent biasing interpretations. This means that the quantitative and qualitative data collection and analysis presented below will be conducted concurrently by the appropriate evaluation team and only brought together during mixed methods data integration. An overview of process evaluation data collection methods alongside trial procedures is presented in Figure 2. Table 1 outlines how the data collection methods described below map onto each research objective.

**Table 1.** Methods to explore intervention implementation and mechanisms of action, including (contextual) factors influencing potential variation.

Construct	Quantitative	Qualitative
**Objective 1 and 2: Implementation of the intervention**		
Fidelity of intervention delivery *consistency of what is implemented with the planned intervention*	• DST analytic data to determine number and length of iFraP sessions and saving/printing of BHR • Patients self-reporting use of the iFraP decision tool and receipt of personalised information in their 2 week and/or 3-month trial questionnaires • Clinician self-reporting printing and/or sending patients their BHR in CRF • Clinician self-reporting sending/providing dentist card in CRF • Fidelity checklist on intervention consult recordings	• Semi-structured interviews with FLS clinicians delivering the intervention and patients randomised to receive the intervention
Dose of intervention delivery *how much of iFraP is delivered*	• DST analytic data to determine number and length of iFraP sessions and saving/printing of BHR • Fidelity checklist on intervention consultation recordings	• Semi-structured interviews with FLS clinicians delivering the intervention and patients randomised to receive the intervention
Adaptions to deliver the intervention *alterations made to iFraP to achieve better contextual fit*	• Fidelity checklist on intervention consultation recordings	• Semi-structured interviews with FLS clinicians delivering the intervention
Barriers and facilitators to intervention delivery		• Semi-structured interviews with FLS clinicians delivering the intervention, patients randomised to receive the intervention, and primary care clinicians
Perceived acceptability of the intervention		• Semi-structured interviews with FLS clinicians delivering the intervention and primary care clinicians
**Objective 3 and 4: Mechanisms of action**		
*FLS clinician mechanisms of action*		
Increased confidence in using DST, communicating risk, health literacy approaches, eliciting and addressing patient preferences and values and SDM	• FLS clinician pre-post eLearning quizzes to gauge FLS understanding of shared decision-making, health literacy, risk communication. • FLS clinician Likert responses in training evaluation forms	• Semi-structured interviews with FLS clinicians delivering the intervention • FLS clinician training evaluation form free-text responses
Identifies appropriate treatment options	• DST analytic data to determine clinician adherence to treatment recommendations produced by the DST	• Semi-structured interviews with FLS clinicians delivering the intervention
*Patient mechanisms of action*		
Greater involvement in decision making	• OPTION 5 observer measure on recorded consultations • Modified Patient-Professional Interaction Questionnaire (PPIQ) in 2 week patient questionnaire • Questions to explore healthcare professional behaviours in FLS appointment in 2-week patient questionnaire	• Semi-structured interviews with FLS clinicians delivering the intervention and patients randomised to receive the intervention
Satisfaction with information/knowledge about condition	• Satisfaction with verbal information in the patient 2-week questionnaire • Satisfaction with written information in the patient 3-month questionnaire • Satisfaction with medicines information in the patient 3-month questionnaire	• Semi-structured interviews with patients randomised to receive the intervention
Realistic expectations of own fracture risk	• Patient self-perceived fracture risk in baseline and 2-week questionnaires compared to actual fracture risk, reported on BHR or calculated using questionnaire data	• Semi-structured interviews with patients randomised to receive the intervention
Illness perceptions: increased perceptions of coherence and controllability	• Modified Brief Illness Perceptions questionnaire in baseline, 2 weeks, and 3-month patient questionnaires • Patient self-report that osteoporosis/bone health was explained to them in the FLS appointment, captured in 2-week questionnaire	• Semi-structured interviews with patients randomised to receive the intervention
Medicine perceptions: Fewer doubts about necessity; lower concerns	• Beliefs about osteoporosis medicines measured in patient 2-week questionnaire • Patient self-reported views of osteoporosis medicines, captured on the BHR and recorded by the FLS clinician during the FLS appointment in the CRF	• Semi-structured interviews with patients randomised to receive the intervention
Increased confidence in decision	• Decisional conflict scale measured in the 2-week questionnaire for patients recommended osteoporosis medicine during their FLS appointment • OPTION 5 observer measure on recorded FLS consultations	• Semi-structured interviews with patients randomised to receive the intervention
Improved decision quality	• OPTION 5 observer measure on recorded FLS consultations	• Semi-structured interviews with patients randomised to receive the intervention
Consistency of messages across social networks, other healthcare settings and FLS	• DST analytic data to determine saving/printing of BHR • Clinician self-reporting completing BHR with patient during the appointment in CRF • Clinician self-reporting sending/providing dentist card in CRF	• Semi-structured interviews with FLS clinicians delivering the intervention, patients randomised to receive the intervention, and primary care clinicians
**Contextual factors that may influence implementation variation (objective 1 & 2) and mechanisms of action (objective 3 & 4)**		
Whether the patient has a bone density scan	• Availability of bone density scan results entered by the FLS clinician in the CRF	• Semi-structured interviews with FLS clinicians delivering the intervention and patients randomised to receive the intervention
Consultation modality (face-to-face or remote)	• Consultation modality captured in FLS completed CRF	• Semi-structured interviews with FLS clinicians delivering the intervention and patients randomised to receive the intervention
Initial prescription given by the FLS	• Medical record review data	• Semi-structured interviews with FLS clinicians delivering the intervention and patients randomised to receive the intervention
Adherence as key performance indicator of services		• Semi-structured interviews with FLS clinicians delivering the intervention
Media and wider social influences on health behaviours		• Semi-structured interviews with FLS clinicians delivering the intervention, patients randomised to receive the intervention, and primary care clinicians

BHR Bone Health Record, CRF case report form, DST Decision Support Tool, FLS Fracture Liaison Service, SDM shared decision-making.

### Consultation recordings

All FLS clinicians (regardless of their allocation to deliver the intervention or usual care) across all four sites will have the opportunity to provide optional consent to audio/video record their consultations with consenting patients. Recordings of iFraP intervention consultations allows intervention implementation to be assessed using a pre-defined fidelity checklist (objective 1). Recording of FLS usual care consultations will examine how risk was discussed and the extent of any contamination.

The observer OPTION 5 scale ^37^ will assess the extent to which clinicians involve patients in the decision-making process about osteoporosis medicines in both trial arms (objective 3). Two raters will independently score a proportion of recordings, guided by the OPTION 5 training manual. ^37^ Ratings will be compared and discussed to assess interrater reliability, followed by further dual scoring, if required. ^38, 39^

### Training evaluation forms

All FLS clinicians allocated to deliver the iFraP intervention completed the iFraP Enhanced Consultation Skills Training Course. To understand the impact of the training course on expected mechanisms of action (objective 3), FLS clinicians were asked to anonymously complete evaluation forms and multiple-choice quizzes at various time points. FLS clinicians were asked to share their views on what was good, what could be improved, any outstanding training needs, and how the course had potential to change their clinical practice, as well as questions to gauge their knowledge. Responses to Likert scales will be summarised using frequencies and proportions and free-text responses will be summarised narratively.

### DST analytics

The iFraP DST will collect analytical data, providing insight into how clinicians use the DST in iFraP intervention consultations (objective 1). Collected analytics include: the length of ‘session’ using the DST, and printing and/or saving of the patient’s personal Bone Health Record. Clinician adherence to treatment recommendations produced by the DST will also be captured (objective 3). Analytical data will be aggregated by FLS site and summarised as frequencies and proportions.

### Case Report Forms

FLS clinicians will complete Case Report Forms (CRFs) hosted on REDCap (a secure web platform for building and managing online databases and surveys) after each iFraP consultation. FLS clinicians allocated to deliver the iFraP intervention will be asked, as part of the CRF, to self-report whether they used the iFraP DST (in full, partially, or not used), if they completed and provided patients with their personal Bone Health Record (yes, printed and handed to the patient; yes, admin to send to the patient at a later date; or not provided) and whether the patient was provided with a dentist card. Clinician responses will be summarised using frequencies and percentages to capture clinician’s self-reported intervention fidelity (objective 1). FLS clinicians delivering the intervention are asked to upload a PDF copy of the patient’s personal Bone Health Record to REDCap. The Bone Health Record includes answers to questions the patient and clinician complete together that aim to capture the patient’s views about medicine benefit and concerns (objective 3).

### Semi-structured interviews

Three participant groups will be invited to take part in a semi-structured interview to explore intervention implementation (objective 1), intervention acceptability (objective 1) and hypothesised mechanisms and outcomes (objective 3, and the influence of contextual factors on this (objectives 1 and 3).

1. Patient participants in the iFraP intervention arm (n = 20)
2. All FLS clinicians delivering the iFraP intervention across all four FLS sites (n = 5–10)
3. Primary care clinicians who consult with a patient following an iFraP intervention consultation (n = 5–10).

Patients that are (1) randomised to receive the iFraP intervention, (2) complete and return their 2-week electronic or paper follow-up questionnaire, and (3) provide consent to be contacted about an interview in the initial trial consent form will be invited to interview. Patients will be purposively sampled to capture variation in characteristics, such as age, sex, FLS site, and decision to take medicine (yes/no/unsure). FLS clinicians delivering the iFraP intervention (iFraP-i), who provide optional consent to take part in an interview, will be invited to take part. Primary care clinicians will be invited to take part in an interview, identified from patients indicating that they visited their general practice post FLS consultation in their 2 week and/or 3-month trial questionnaires or during their semi-structured interview. Primary care clinicians will be aware about the possibility of being contacted for interview in the letter sent to notify them of their patient’s participation in the trial.

All interviews will be completed in-person, by telephone, or using Microsoft Teams. Interview topic guides are developed informed by findings of the iFraP in-practice feasibility testing ^40^ and hypothesised programme theory. The topic guides will also be sensitised using the Normalisation Process Theory (NPT) to focus on the ‘work’ that individuals and groups do to enable intervention implementation. ^41^ The four key NPT components are: coherence (or sense-making); cognitive participation (or engagement); collective action (work done to enable the intervention to happen); and reflexive monitoring (formal and informal appraisal of the benefits and costs of the intervention). Topic guides will be iteratively updated, based on consultation recordings, ongoing interview findings, and discussions with expert CoP stakeholders and public contributors.

Interviews will be recorded and transcribed verbatim. Data will be inductively analysed using the framework method to develop an analytical framework to systematically apply to the dataset ^42^ using NVivo data management software. Use of a ‘matrix’ will help to reduce the data in order to analyse it ‘by case’ and ‘by code’, supporting interdisciplinary and collaborative discussions with team members, CoP and public contributors. The NPT will facilitate data interpretation to unpick factors that promoted and inhibited implementation of the iFraP intervention during the randomised controlled trial, and help to think through intervention implementation post-trial.

### Patient trial data collection

Patients participating in the trial are asked to complete questionnaires at baseline (before their FLS appointment) and 2 weeks and 3 months after their FLS appointment. Questionnaires will be completed online or by paper, depending on the patient’s preference. The hypothesised mechanisms and outcomes of the iFraP intervention, as detailed in the iFraP programme theory, informed the choice of outcome measures included in patient questionnaires (objective 3). Shared decision-making was a key hypothesised mechanism underpinning the iFraP intervention, including concepts such as: patient self-reported ease in decision-making (Decisional Conflict Scale ^43^), patient centred care (Patient-Professional Interaction Questionnaire [PPIQ] ^44^), illness and treatment perceptions (modified Brief Illness Perceptions Questionnaire [BIPQ] ^45^ and Beliefs about Medicines Questionnaire [BMQ] ^46^), and satisfaction with information about bone health (modified satisfaction with cancer information profile [SCIP] ^47^). Additional hypothesised outcomes, such as medicine initiation and persistence will also be captured in patient self-reported questionnaires. A complete list of outcome measures included in the patient questionnaires are provided in the trial protocol. ^27^

Patient questionnaires will also ask the patient participant to report:

• whether the FLS clinician implemented ‘essential’ tasks in the FLS consultation (objective 1). ‘Essential’ consultation tasks (e.g. ‘to what extent did the healthcare professional explain the aim and purpose of the appointment?’) were determined by the iFraP intervention development consensus survey ^48^ and integrated into the Enhanced Consultation Skills Training Course and underpinned the iFraP DST structure.
• if they received the intervention in their FLS appointment (2 week questionnaire) or personalised written information during/after the FLS appointment (3 month questionnaire) to investigate intervention implementation (objective 1).

Data will be summarised and reported as frequencies and percentages, means and standard deviations, or medians and inter-quartile ranges, as appropriate. Statistics (such as t-tests, correlations, chi-square tests) will be used to explore relationships in the data (e.g. to compare between trial arms) to test and refine the programme theory. Additional exploratory statistical analysis may be conducted, where appropriate, guided by emerging findings from the qualitative interviews and other process evaluation methods. We will also consider contextual factors that may play a role in implementation variation, for example by stratifying results by FLS sites that deliver face-to-face vs telephone consultations.

### Integrating findings

It is common for qualitative research to be conducted alongside randomised controlled trials to evaluate complex interventions. However, quantitative and qualitative findings are rarely integrated. ^49^ A triangulation protocol will be used to integrate the quantitative and qualitative findings to generate novel insights about the intervention under evaluation. ^50^ Key finding statements from each dataset will be displayed in a convergence coding matrix ^49, 51^ to assess agreement (consistent findings), partial agreement (some overlap or consistency between findings, but also some differences or gaps), silence (data source does not provide information relevant to specific finding), or dissonance (conflicting or contradictory findings) between findings. Team members from the process and outcome evaluation teams, wider study team members, CoP stakeholders, and public contributors will facilitate interpretations of ‘agreement’ between data sources and development of ‘meta-themes’ (themes that cut across the findings from different methods), thereby enhancing rigour and credibility. ^52^

## Data management

All information collected during the trial and process evaluation will be kept strictly confidential. An external company will complete transcription with confidentiality and data sharing agreements in place. When returned to the research team, identifiable characteristics (e.g. names, places) will be removed from the transcript and recording files stored on the protected secure network.

At the end of the trial and process evaluation, data will be securely archived in line with the Sponsor’s procedures for a minimum of 10 years. Data held by Keele CTU will be archived in the designated Keele CTU archive facility. Following authorisation from the Sponsor, arrangements for confidential destruction will then be made.

Any subsequent requests for access to the data (e.g. collaboration, joint publication, data sharing requests from publishers) will follow Keele University’s standard operating procedure. Further information can be found in the trial protocol. ^27^

## Conclusion

This paper describes the design of a mixed methods process evaluation, running in parallel with the iFraP pragmatic randomised controlled trial, to understand what elements of the iFraP intervention are implemented and how, how the delivered iFraP intervention produces change (or not), and how context affects implementation of iFraP and outcomes. The process evaluation findings will add to the existing evidence base examining DST implementation, ^16, 18^ with novel contributions about the role of complementary enhanced clinician training to facilitate shared decision-making and the delivery of shared decision-making and DSTs in consultations delivered using varied modalities, including remote consultations. ^25^

## Ethics

Ethical approval for this process evaluation is incorporated into the ethics application for the iFraP randomised controlled trial. Ethical approval was obtained from East of Scotland Research Ethics Service (EoSRES) (22/ES/0038) on 21 ^st^ October 2022.

Participants will give full informed written consent to data collection from interviews, consultation recordings, CRFs, and trial questionnaires. Clinician anonymous training evaluation data was provided under implied consent. The approach to consent was approved by the ethics committee.

Following initial approval from the Research Ethics Committee (REC), the REC will be continually informed of all substantial changes to the management of the study. Routine reporting will take place in line with REC requirements.

## Trial sponsor

Trial Sponsor: Keele University.

Sponsor’s Reference: RG-0345-22.

## Data Availability

No data are associated with this article. Not applicable.
