# Supplementary material for: Protocol for a mixed methods process evaluation for a randomised controlled trial to improve shared decision-making about, and uptake of, osteoporosis medicines: the iFraP study
Source: NIHR Open Res. 2026 Apr 2;4:70. Originally published 2024 Nov 13. [Version 3] doi: 10.3310/nihropenres.13751.3 (PMC12743211; doi:10.3310/nihropenres.13751.3)
Supplement: Peer review history

# Reviewer report: Reviewer response for version 2

**DOI:** 10.3310/nihropenres.15372.r38441

**Published:** 2025-12-26

**Authors:** Martina Behanova

**Affiliations:** 1 Ludwig Boltzmann Institute of Osteology At Hanusch Hospital of OEGK and AUVA Trauma Center Meidling 1st Med.Dep. Hanusch Hospital Heinrich Collin Str. 30 1140 Vienna, Austria, Vienna, Austria

The iFraP protocol is a strong, well-designed and clinically relevant study with real potential to advance shared decision-making and improve osteoporosis treatment rates. I particularly value the involvement of public contributors with osteoporosis and carers throughout development, data interpretation and dissemination, as well as the intention to examine delivery across both face-to-face and remote consultations. Even though the design is robust, I think it could be strengthened by considering a few points related to fidelity, clinician motivation, patient understanding and equity.

 Regarding fidelity, it will be documented comprehensively, but mainly in terms of whether tasks were completed rather than whether patients actually understood them. In my view, the consultation recordings are the most powerful fidelity tool in the study, yet they may be under-used as currently planned. Currently, it measures delivery, but not absorption. Adding teach-back analysis, where patients demonstrate understanding of their fracture risk or treatment rationale, would meaningfully differentiate true shared decision-making from procedural box-ticking.

 Clinician motivation is another area that warrants deeper attention. While the protocol will capture clinician behavior through evaluation forms, quizzes, case report forms and decision support tool (DST) analytics, it does not currently assess clinician belief or readiness as a potential predictor of fidelity. Shared decision-making (SDM) depends on whether clinicians genuinely believe it is beneficial, efficient, and aligned with their professional identity, motivation may strongly influence how consistently iFraP is delivered in practice. A brief baseline and follow-up assessment of attitudinal commitment to SDM could help explain variation in delivery and better predict sustainability beyond the trial. This would complement the Normalisation Process Theory-informed interview work already planned and strengthen the link between observed behaviour and underlying drivers.

 The intervention requires longer consultations. While this is manageable within the four participating UK FLS sites, scalability to busier or less well-resourced services — particularly where IT infrastructure or staffing is more variable — remains an important question for future implementation work.

 Equity has been acknowledged, but with 20 participants per arm subgroup analysis is not possible, so the trial will not be able to tell whether the intervention benefits all groups to the same extent.

 The protocol is transparent about what data will be collected but less clear on how those data will be organised, managed, and made analytically usable. Several questions remain, particularly for the qualitative component: How will qualitative data be coded, and according to which framework? How will interviews be anonymised, stored, and processed, and which software will be used? Likewise, it is not yet explained how terms within the convergence matrix— *agreement, partial agreement, silence,* and *dissonance*—will be defined operationally or resolved in practice. It would also be valuable to clarify data security procedures and outline whether data will be shared or deposited in a long-term repository, as this will influence reproducibility and secondary analysis.

 Overall, the authors present a well-conceived study with a clear rationale, thoughtful methodology and appropriate integration of quantitative and qualitative work. The process evaluation is well matched to the complexity of SDM, and the triangulation approach should uncover how the intervention is applied in practice rather than whether it works in isolation.

 My comments are intended only to enhance the richness of interpretation. With minor refinement — particularly in how clinician motivation and patient comprehension are captured — iFraP has the potential not only to improve treatment uptake within FLS pathways but also to contribute fundamentally to our understanding of what meaningful shared decision-making looks like in routine care. I look forward to seeing the results of this important study and the insights it will bring to osteoporosis management and to SDM implementation science more broadly.

Is the study design appropriate for the research question?

Yes

Is the rationale for, and objectives of, the study clearly described?

Yes

Are sufficient details of the methods provided to allow replication by others?

Yes

Are the datasets clearly presented in a useable and accessible format?

Not applicable

Reviewer Expertise:

public health, clinical epidemiology, registry-based research, data analysis and statistical modelling

I confirm that I have read this submission and believe that I have an appropriate level of expertise to confirm that it is of an acceptable scientific standard.

# Response

**Published:** 2026-02-24

**Authors:** Laurna Bullock

**Affiliations:** School of Medicine, Keele University, Stoke on Trent, England, UK

Thank you for providing thoughtful review of the iFraP process evaluation protocol. Below, we have outlined each of your comments, followed by a response.

**Reviewer comment:** The iFraP protocol is a strong, well-designed and clinically relevant study with real potential to advance shared decision-making and improve osteoporosis treatment rates. I particularly value the involvement of public contributors with osteoporosis and carers throughout development, data interpretation and dissemination, as well as the intention to examine delivery across both face-to-face and remote consultations. Even though the design is robust, I think it could be strengthened by considering a few points related to fidelity, clinician motivation, patient understanding and equity.

**Author response: **Thank you for your positive comments and spending the time to provide thoughtful feedback. Data collection for the iFraP randomised controlled trial and process evaluation finished in November 2024 and analysis is currently being finalised. We therefore acknowledge our limited scope to make larger changes suggested by the reviewer. Specifically, we are unable to amend the study data collection methods at this point and will therefore consider the points raised below as study limitations when we come to write the final results paper.

**Reviewer comment:** Regarding fidelity, it will be documented comprehensively, but mainly in terms of whether tasks were completed rather than whether patients actually understood them. In my view, the consultation recordings are the most powerful fidelity tool in the study, yet they may be under-used as currently planned. Currently, it measures delivery, but not absorption. Adding teach-back analysis, where patients demonstrate understanding of their fracture risk or treatment rationale, would meaningfully differentiate true shared decision-making from procedural box-ticking.

**Author response: **Thank you for this comment. We are using a variety of methods (e.g. DST analytics, consultation recordings, patient self-reporting use of DST in trial surveys, case report forms and qualitative interviews) to gain a multi-dimensional understanding of implementation variation, its impact on mechanisms of action, outcomes, and the role of context. This means that we did not pre-define thresholds of “acceptable” fidelity/dose. We agree that the audio recorded consultations focus on intervention delivery rather than patient knowledge or understanding. Patient self-reported questionnaires (including measures of decisional conflict, satisfaction with information, illness and treatment perceptions, and fracture risk perception) and semi-structured interviews aim to explore patient perceptions of shared decision-making, including their understanding or perception of information provided during the consultation (e.g.their fracture risk).

**Reviewer comment:** Clinician motivation is another area that warrants deeper attention. While the protocol will capture clinician behavior through evaluation forms, quizzes, case report forms and decision support tool (DST) analytics, it does not currently assess clinician belief or readiness as a potential predictor of fidelity. Shared decision-making (SDM) depends on whether clinicians genuinely believe it is beneficial, efficient, and aligned with their professional identity, motivation may strongly influence how consistently iFraP is delivered in practice. A brief baseline and follow-up assessment of attitudinal commitment to SDM could help explain variation in delivery and better predict sustainability beyond the trial. This would complement the Normalisation Process Theory-informed interview work already planned and strengthen the link between observed behaviour and underlying drivers.

**Author response: **Thank you for this comment, we agree that pre-and-post assessments would have provided useful insights into clinician perceptions of delivering the intervention. Instead, training course evaluation forms and qualitative interviews completed by clinicians aim to understand perceptual (e.g. motivation, readiness) and practical (e.g. skills, resources) barriers and enablers to intervention delivery and sustainability, whilst also considering complex contextual factors.

**Reviewer comment:** The intervention requires longer consultations. While this is manageable within the four participating UK FLS sites, scalability to busier or less well-resourced services — particularly where IT infrastructure or staffing is more variable — remains an important question for future implementation work.

**Author response: **Thank you for this comment. A previous Cochrane review [1] identified that in-consultation decision aids increased the length of consultation by 1.50 minutes compared to usual care. In this process evaluation, the length of consultations will be obtained from DST analytics (for intervention consults) and consultation audio recordings (for intervention and usual care consults). This data will facilitate understanding of whether the iFraP intervention increased the length of the consultation. Practical enablers and barriers to intervention implementation, both within and beyond the trial, will be explored with clinicians in interview.   

1. Smith, M., Carley, M., Volk, R., Douglas, E. E., Pacheco-Brousseau, L., Finderup, J., ... & Trevena, L. (2024). Decision aids for people facing health treatment or screening decisions. *Cochrane Database of Systematic Reviews*, (1).

**Reviewer comment:** Equity has been acknowledged, but with 20 participants per arm subgroup analysis is not possible, so the trial will not be able to tell whether the intervention benefits all groups to the same extent.

**Author response: **The iFraP randomised controlled trial aimed to recruit 380 patient participants randomised (1:1 ratio) to the intervention or usual care who will complete self-report questionnaires at baseline and follow up at 2-weeks and 3-months. This will allow for subgroup analysis to if and how patient and site characteristics lead to variation in outcomes. A small portion of participating trial patients will complete an interview (approx. n=20) to explore these variations in more detail.

**Reviewer comment:** The protocol is transparent about what data will be collected but less clear on how those data will be organised, managed, and made analytically usable. Several questions remain, particularly for the qualitative component: How will qualitative data be coded, and according to which framework? How will interviews be anonymised, stored, and processed, and which software will be used? Likewise, it is not yet explained how terms within the convergence matrix— *agreement, partial agreement, silence,* and *dissonance*—will be defined operationally or resolved in practice. It would also be valuable to clarify data security procedures and outline whether data will be shared or deposited in a long-term repository, as this will influence reproducibility and secondary analysis.

**Author response: **The qualitative data will be analysed using the commonly used ‘framework method’. The analytical framework will be developed inductively, and then systematically applied to the dataset using NVivo data management software. An external company will complete transcription with confidentiality and data sharing agreements in place. When returned to the research team, identifiable characteristics (e.g. names, places) will be removed from the transcript and recording files stored on the secure network. Trial and process evaluation data will not be shared or deposited in a long-term repository. Release of data will be subject to a data use agreement between the Sponsor and the third party requesting the data. Further information about data storage is provided in the trial protocol paper: https://doi.org/10.1186/ISRCTN10606407 and the ethically approved protocol published on the ISRCTN: https://doi.org/10.1186/ISRCTN10606407. We have added detail to the manuscript about data management processes.

 The convergence matrix will display findings from each data collection method. The matrix will be used to assess agreement (consistent findings), partial agreement (some overlap or consistency between findings, but also some differences or gaps), silence (data source does not provide information relevant to a specific finding or outcome), or dissonance (conflicting or contradictory findings). Partial agreement or dissonance will be embraced as opportunities for deeper insight rather than errors to be ‘resolved’. To validate judgements of ‘agreement’ and to explain dissonance (if present), we will work collaboratively with the multidisciplinary team, including public contributors, and triangulate our findings with external evidence. We have updated the manuscript to provide definitions of terms (e.g. ‘convergence’, ‘silence’) and how we will operationalise these terms to ensure validity and credibility.

# Reviewer report: Reviewer response for version 2

**DOI:** 10.3310/nihropenres.15372.r37967

**Published:** 2025-11-21

**Authors:** Zaid Al-Sawaff

**Affiliations:** 1 Northern Technical University, Mosul, Iraq

This is a **process-evaluation protocol** to run alongside a pragmatic RCT of the iFraP intervention in UK Fracture Liaison Services (FLS). The intervention combines a decision-support tool, enhanced consultation training, and patient materials to improve medicines decision-making in osteoporosis. The protocol specifies a mixed-methods design: quantitative components (case-report forms, patient questionnaires, decision-support usage analytics) and qualitative components (interviews, consultation recordings), with a pre-planned **triangulation** approach to integrate findings. It is grounded in an explicit programme theory and established implementation frameworks, includes ethics approvals and trial registration, and outlines Patient and Public Involvement (PPI).

 Major comments

1. The protocol lists measures of fidelity (e.g., DST use, OPTION-5 ratings, decisional measures) but does not pre-define **a priori thresholds** for what constitutes “acceptable fidelity/dose” or a **minimally important difference** on key scales (e.g., OPTION-5, Decisional Conflict Scale).
2. You plan to sample usual-care consultations, but it is not clear **how contamination** (i.e., spill-over of intervention elements) will be quantified and modelled.
3. Purposive sampling is appropriate, but **target numbers by role/site**, stopping rules for **data saturation**, and management of cross-site heterogeneity need more precision.
4. A convergence matrix is mentioned, but the **rules for resolving dissonance** between qualitative and quantitative strands are not specified.
5. The protocol notes face-to-face vs remote delivery, but **equity analyses** (digital access, health literacy) and **missing-data plans** across modes are not pre-specified.

Is the study design appropriate for the research question?

Yes

Is the rationale for, and objectives of, the study clearly described?

Yes

Are sufficient details of the methods provided to allow replication by others?

Partly

Are the datasets clearly presented in a useable and accessible format?

Not applicable

Reviewer Expertise:

biomedical engineering, medical instrumentation techniques , computational chemistry

I confirm that I have read this submission and believe that I have an appropriate level of expertise to confirm that it is of an acceptable scientific standard, however I have significant reservations, as outlined above.

# Response

**Published:** 2026-02-24

**Authors:** Laurna Bullock

**Affiliations:** School of Medicine, Keele University, Stoke on Trent, England, UK

Thank you for providing thoughtful review of the iFraP process evaluation protocol. Below, we have outlined each of your comments, followed by a response.

**Reviewer comment:** The protocol lists measures of fidelity (e.g., DST use, OPTION-5 ratings, decisional measures) but does not pre-define a priori thresholds for what constitutes “acceptable fidelity/dose” or a minimally important difference on key scales (e.g., OPTION-5, Decisional Conflict Scale).

**Author response: **Thank you for your comment. Intervention implementation (fidelity and dose) will be explored using various methods, including: DST analytics, consultation recordings, patient self-reporting use of DST in trial surveys, case report forms and qualitative interviews. The exploratory approach means that we did not pre-define thresholds of “acceptable fidelity/dose”. Instead, we will explore implementation variation, its impact on outcomes, and the role of context.

 The OPTION-5 and Decisional Conflict Scales are measures of shared decision-making and will not be used to make conclusions about intervention implementation. Minimally important differences are not defined for these measures.

**Reviewer comment:** You plan to sample usual-care consultations, but it is not clear how contamination (i.e., spill-over of intervention elements) will be quantified and modelled.

**Author response: **A predefined checklist will be used to record and quantify the presence of intervention components in the intervention and usual care consultation recordings. Use of the predefined checklist will allow for numerical comparison of any possible contamination between the trial arms.

**Reviewer comment:** Purposive sampling is appropriate, but target numbers by role/site, stopping rules for data saturation, and management of cross-site heterogeneity need more precision.

**Author response: **Patients who received the intervention in their FLS appointment will be purposively sampled in an iterative manner to capture maximum variation in a range of characteristics: age, sex, FLS site, and decision to take medicine (yes/no/unsure). By sampling patients across each of the four participating FLS sites, we aim to capture ‘cross-site heterogeneity’ – this is important when considering how contextual (e.g. site-specific) factors may influence implementation, mechanisms of action, and outcomes. We did not define strict ‘stopping rules’ for data saturation – instead, interviews will continue until the research team judge that the data provided sufficient interpretive richness to address the research questions.

**Reviewer comment:**  A convergence matrix is mentioned, but the rules for resolving dissonance between qualitative and quantitative strands are not specified.

**Author response: **It is possible that the convergence matrix will identifying dissonance between quantitative results and qualitative findings. Dissonance (or ‘conflicting’ findings) should be embraced as opportunities for deeper insight rather than errors to be resolved. If dissonance is identified during integration, it is good practice to review the data quality, reliability, and validity. If dissonance cannot be attributed to methodological limitation, additional exploration of collected data, collaboration with the multidisciplinary team, and previous literature will validate our conclusions. We have updated the manuscript to provide definitions of terms (e.g. ‘convergence’, ‘silence’) and how we will operationalise these terms to ensure validity and credibility.

# Reviewer report: Reviewer response for version 1

**DOI:** 10.3310/nihropenres.14938.r33785

**Published:** 2025-01-23

**Authors:** Peter R. Ebeling

**Affiliations:** 1 Monash University, Clayton, Victoria, Australia

"Bullock L et al. Protocol for a mixed methods process evaluation for a randomised controlled trial to improve shared decision-making about, and uptake of, osteoporosis medicines: the iFraP study”.

 This is a well-constructed and detailed protocol for a mixed methods process evaluation for a randomised controlled trial to improve shared decision-making about, and uptake of, osteoporosis medicines (iFRAP). This is an important area as although osteoporosis medications are both effective and cheap, both treatment initiation and persistence at 12 months are low, even when patients are seen in a fracture liaison service, the best standard of care. 

 Quantitative data will be collected using case report forms; self-reported questionnaires completed by patients; and decision support tool analytic data. Qualitative data will be collected using semi-structured interviews with patients receiving the iFraP intervention, FLS clinicians delivering iFraP appointments, and primary care clinicians consulting with a patient after their iFraP FLS appointment; and FLS consultation recordings. The quantitative and qualitative findings will be integrated to generate novel insights about the intervention. The process evaluation, alongside the trial, will help to understand the effectiveness and acceptability of the iFraP intervention. Other insights about the implementation of shared decision making and the use of decision support tool in practice will be gained.

 The study design is well conceived and robust without any areas of concern on reducing feasibility. If effective it could lead to changes in clinical practice in osteoporosis management, particularly pertaining to fracture liaison services.

 This reviewer has no comments for further enhancements or changes to the protocol.

Is the study design appropriate for the research question?

Yes

Is the rationale for, and objectives of, the study clearly described?

Yes

Are sufficient details of the methods provided to allow replication by others?

Yes

Are the datasets clearly presented in a useable and accessible format?

Yes

Reviewer Expertise:

Endocrine disorders

I confirm that I have read this submission and believe that I have an appropriate level of expertise to confirm that it is of an acceptable scientific standard.

# Response

**Published:** 2025-10-09

**Authors:** Laurna Bullock

**Affiliations:** School of Medicine, Keele University, Stoke on Trent, England, UK

Thank you to the reviewer for their positive feedback about the iFraP mixed methods protocol.
